# Evidence-Based Strategies for Reforming the Medical Curriculum at the National Autonomous University of Honduras: A Systematic Literature Review

**DOI:** 10.7759/cureus.68729

**Published:** 2024-09-05

**Authors:** Génesis S Henriquez, Fernando J Caceres Carranza, Kristopher J Varela, Julia C Salinas Ulloa, Rossana Reyes, Jhiamluka Solano

**Affiliations:** 1 Medicine, Asociación de Educación Médica Hondureña, Tegucigalpa, HND; 2 Hospitalization, Roatan Hospital, Roatan, HND; 3 General Practice, Faculty of Medical Sciences, Universidad Nacional Autónoma de Honduras, Tegucigalpa, HND; 4 Medical Education, Asociación de Educación Médica Hondureña, Tegucigalpa, HND; 5 General Medicine, Universidad Nacional Autónoma de Honduras, Tegucigalpa, HND; 6 General Practice, Universidad Nacional Autónoma de Honduras, Tegucigalpa, HND; 7 Medicine, Universidad Nacional Autónoma de Honduras, Tegucigalpa, HND; 8 Internal Medicine, Universidad Nacional Autónoma de Honduras, Tegucigalpa, HND; 9 Cardiology, Scunthorpe General Hospital, Scunthorpe, GBR; 10 Education, Academy of Medical Educators, Cardiff, GBR

**Keywords:** developing countries, integrated curriculum, medical curricular reform, medical education challenges, patient-centred care

## Abstract

Medical education worldwide has undergone numerous stages of reform. Cultural and financial restraints have decelerated progress in developing countries. Current reforms should focus on creating integrated, competency-based, and student-centered curricula that emphasize patient-centered care. The following review of literature published between 2014 and 2023 on global curricular reforms highlighted key components, challenges, and strategies for implementing or evaluating undergraduate medical programs that prioritize student-centered approaches and competency-based models. This review also compared the current curriculum at the National Autonomous University of Honduras (UNAH) with these international experiences to suggest strategies in order to encourage significant reform. The following review identified 47 articles that provided insights into ideal contexts for curricular reforms, while 15 publications detailed the current state of the UNAH medical curriculum and its potential weaknesses. Additionally, 25 articles discussed specific reforms in other countries, offering valuable results and conclusions for consideration. Drawing from these models and experiences, strategies were proposed for UNAH’s curriculum reform, including identifying basic needs, defining project vision, training teaching staff and students, and integrating multidisciplinary teams of experts. Although training all teaching staff abroad may be financially unfeasible, selecting and training key individuals to train others could be a viable alternative. Successful reform requires a comprehensive, periodic, and systematic evaluation. Despite the challenges faced by developing countries, global experiences with alternative reform models offer promising solutions, providing an opportunity for the Faculty of Medical Sciences at UNAH to overcome local limitations and fulfill the primary task of training professionals who are clinically, ethically, and adaptively competent, with a focus on patient-centered and primary care approaches.

## Introduction and background

Higher education systematically prepares students to acquire the skills necessary to perform a profession. Within this, we find a branch known as medical education. This focuses on the integration of knowledge appropriate for the clinical environment and the management of patients in a holistic manner. The purpose of medical curriculums should be to transmit knowledge to future professionals, forming skills and instilling values ​​in a balanced and integrated manner to ensure quality care. It is important to distinguish that, although medical education derives from higher education, the teaching methodologies that must be used are divergent since they fulfill specific functions. Furthermore, the speed with which health and medical education evolves demands timely curricular reforms.

The Lancet Commission in 2010 suggested moving from the sequential approach from basic and preclinical to clinical sciences, adopting problem-based learning (PBL), and the need to implement an approach based on professional competencies by addressing systems and organs of local relevance without undermining global knowledge. Medical schools in developing countries have been left behind in curricular reform processes with scientific support. To date, there is no evidence to support traditional systems that have been in place for some decades. Similarly, there is abundant evidence that the final product delivered to society is not ideal.

The last reform to the medical curriculum of the National Autonomous University of Honduras (UNAH) was carried out in 1995. It also had a process of reorganization and systematization of execution in the year 2000. During this, the concept of competencies and their levels were discussed superficially. The syllabus provides a list of the content of the subjects, but it does not specify the expected level of competence that the doctor requires before graduating and practicing medicine without direct supervision. Furthermore, there is no record of the use of modern teaching, integration, or assessment methodologies supported by scientific evidence. Due to this, there is a knowledge gap around the training processes in our faculty. Therefore, a review of the literature was carried out to identify strategies used by other countries during their curricular reforms that can be used or adapted to our cultural and academic environment. This in order to carry out a reform to the medical curriculum with student-centered learning approaches based on clinical and professional competencies.

Methodology

Search Strategy

A systematic review of the literature on medical education reforms was conducted in PubMed. We used "Medical Education" and "Reforms" as search terms. Literature review articles, perceptions, experiences, and systematic reviews published in English or Spanish between 2014 and 2023 were included. However, articles published before 2014 were included to provide local context to enhance the development of the research question (33,41,44,48,49,50,51,52,54,56,60,71,85). The search strategy aimed to cast a wide net while ensuring accuracy, with an emphasis on capturing studies that closely align with the research objectives. The authors then selected articles based on title, summary, and full articles.

Inclusion Criteria

These criteria were predefined based on the research question and objectives. Among them are articles that expose the experience of undergraduate medical curricular reform in other countries with student-centered approaches, based on competencies and integrated models.

Exclusion Criteria

Articles that were not original studies, book chapters, letters to the editor, abstracts, articles published in languages ​​other than English or Spanish, not related to medical education, medical postgraduate reforms, and outside were excluded. Studies that did not meet these criteria were systematically excluded from consideration, thus improving the quality and reliability of the synthesized evidence.

Study Selection

The study selection process involved a systematic approach to select and evaluate potentially eligible studies identified through the search strategy. Initially, titles and abstracts were compared against predefined inclusion and exclusion criteria to identify relevant articles for full-text review. Full-text articles were then assessed in detail to determine their suitability for inclusion based on established criteria.

Data Extraction

Once study selection was completed, a structured approach was used to extract relevant data from the included studies. Using Zotero as a bibliographic manager, data were extracted systematically. Key information includes the characteristics of the study, the participants, and the results or conclusions of the intervention.

Analysis of Data

Quantitative data were synthesized by appropriate subgroups, while qualitative data were analyzed thematically or through content analysis, as appropriate, extracting significant patterns and ideas.

Results

**Figure 1:**
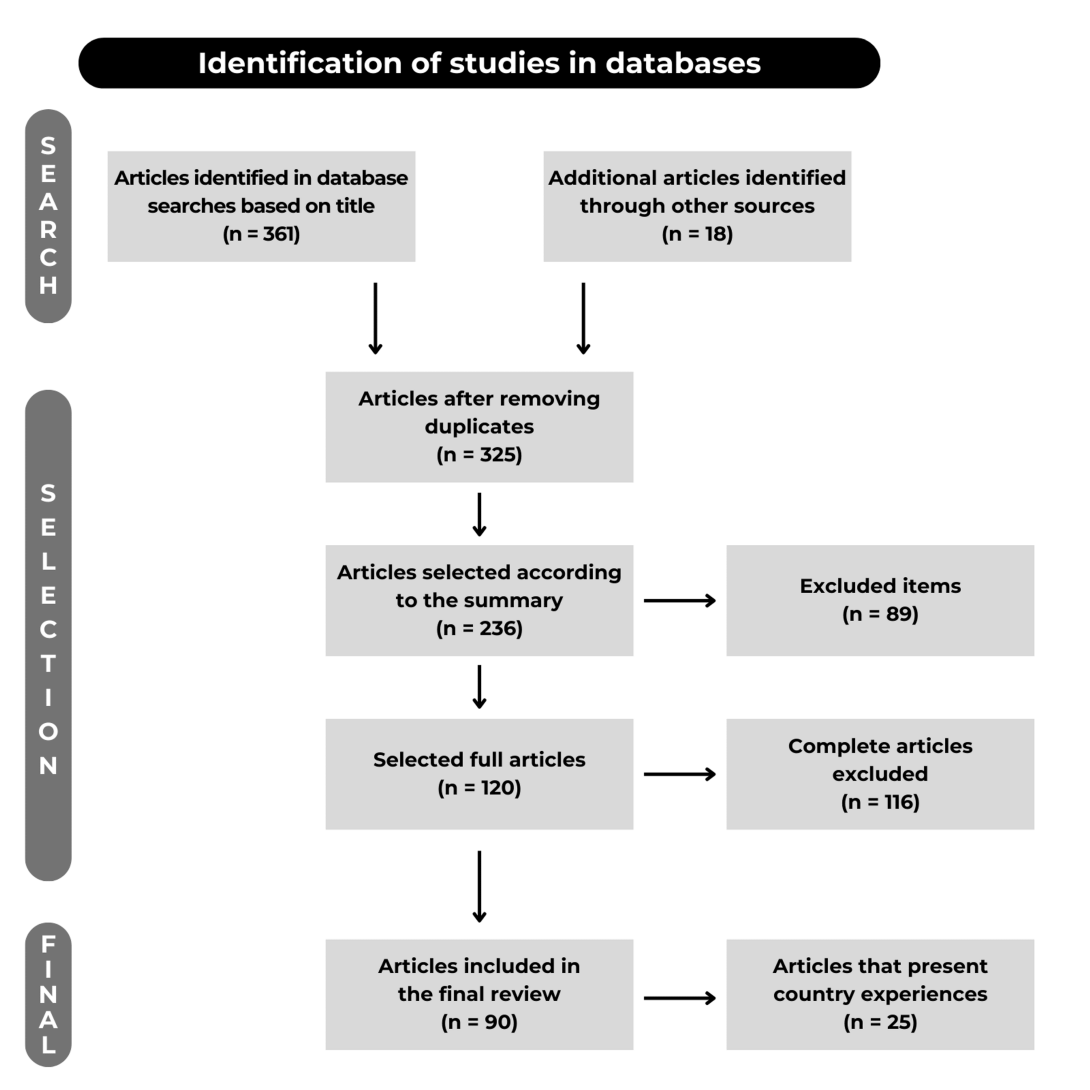
PRISMA diagram. PRISMA: Preferred Reporting Items for Systematic Reviews and Meta-Analyses

The search, identification, selection, and review process is shown as a Preferred Reporting Items for Systematic Reviews and Meta-Analyses (PRISMA) diagram (see Figure 1). The articles were addressed in three different stages. The first consisted of selecting titles and abstracts, which helped identify a list of articles that could answer the research question. The second consisted of screening the full text of the selected articles to discard articles that do not include data relevant to the research. Finally, the third stage consisted of filtering the references of the selected articles to find more articles that were potentially useful to develop the review, the last stage is also known as the snowball method and is used to identify valuable and relevant secondary references that are presented in the main articles reviewed. The snowball method was advantageous in topics where it was difficult to find many publications to improve the quality of the review.

## Review

Comprehensive curriculum

The Edinburgh Declaration (1988) established the process of formal expansion of medical education through integration, student-centered learning, evidence-based medicine, professionalism, and ethics [1,2]. Among the goals for curricular plans, they emphasize acquiring medical knowledge based on reflective practice, interpersonal skills, taking responsibility, and professionalism [3,4]. Remember that learning does not end upon graduation but is a permanent process influenced by the training [5]. The Lancet Commission distinguishes three levels of education (see Table 1) [6].

**Table 1:** Levels of knowledge described by the Lancet Commission. Ref. [6]

Education Levels	Description
Informative	Focused on knowledge
Formative	Focused on the development of professionalism
Transformer	Student-centered to act as an agent of change

After the Flexner reform, medical educators realized that factual knowledge of the unattainable expectation of absolute memory was not enough to train competent doctors, and it became imperative to focus on the clinical relevance of why and when the insights would be useful [7-11]. Overloading the student with details of little clinical importance denotes a distorted perception of teaching [7]. Emphasis must be on the use of clinically oriented methodologies, fostering understanding through research and simulation since it can bridge the gap between theory and practice and empower areas that have been underestimated such as immunology, radiology, orthopedics, and psychiatry [12-16].

Active Learning

The teacher-centered approach reduces the critical thinking, adaptation capacity, participation, and attendance of students [6,17]. The first step to active learning is for students to identify their learning style [18]. Although the responsibility of learning mostly involves the student, the fundamental role of the teacher in moderating the content should never be underestimated [19].

Competency-Based Learning

The Universal Declaration of Human Rights (1948) emphasized that health systems must ensure that health providers are competent [20]. Decades after the Flexner reform, poor quality health care and non-standardized learning became noticeable [21]. Not defining what level is expected of the student generates varied expectations, and this subjective uncertainty leads to stress and anxiety [22]. Competences, beyond the theoretical-clinical field, must also include professional skills [23].

Student-Centered Learning

Delimiting competencies is simply the first step, and the second is allowing individualized learning trajectories to prepare students for independent clinical practice [24,25]. Student perception is key to engagement; therefore, making the student the focus of learning will improve its impact [26].

Patient-Centered Care

It is believed that a doctor does not need to be a humanist to be successful [27]. It is curious that countries that focus on standardized testing are at the bottom of healthcare quality [21]. A model with new educational standards prepares the student to serve the needs of society [21,28]. Professional competencies cover communication, reflection, clinical reasoning, psychology, emotion management, teamwork, and leadership with a social, humanistic, and ethical approach [29-31]. The simulation allows the student to have a doctor-patient interaction in an environment where they reflect on behaviors prior to interacting with patients [32].

Accreditation

An accreditation process is necessary; the Central Committee of the Institute for International Medical Education defined the global essential competencies required by health professionals, known as the “Global Minimum Essential Requirements” [4]. These must be corroborated by medical schools so that students can obtain their degrees [33]. The accreditation process is a self-assessment process, subsequently addressed by an external evaluation visit that provides recommendations to improve the indicators [34].

Challenges of an innovative reform

Participation and Acceptance of Innovation

Many teachers are aware that their efforts do not generate meaningful learning, but they still do not encourage change [35]. Frustration should be anticipated when asking educators to transform the methods they have always used [17]. Scientifically, teachers and students must adopt a radical philosophy, accepting that the ideal may be far from what they previously practiced [36]. Transformational leadership that takes risks must be promoted [37]. The reform should not be imposed, but rather a collaborative approach involved from the early stages to avoid mistrust and lack of collaboration [9,30].

Resource Availability

Developing countries have greater needs but fewer resources [20]. The economic situation in medical schools is based on a tight budget that makes the reform unviable [38]. With timely training, teachers can adapt methodologies even with a low budget [39]. Rejuvenating processes lead to appropriate reform [4]. The hiring of academics whose sole focus is curricular reform is suggested; the participation of international experts, auditors, or external evaluators is encouraged, remembering that successful reform will remain in continuous improvement [35].

Social, Economic, and Cultural Environment

Historical and cultural events in a country can lead to delays in innovation [40]. The key to reform is considered to be the transformation of culture [41]. The overload of patients of clinical teachers and a large number of students prevent teaching and evaluating efficiently [23]. If the need for change is recognized but not implemented, medical schools must approach the question of 'why change has been so difficult?' and address that resistance [9].

Specialized and Updated Qualification

There exists limited guidance on appropriate formative processes for educators that might promote problematic practices [42]. Teachers express discomfort about the reforms due to the greater effort and work required for the transition [43]. Topics must be discussed frequently and deeply with students for competency-based training and evaluation [44]. In a clinically successful teacher-student relationship, the teacher must see the student as an equal partner; unfortunately, the teachers might prioritize maintaining hierarchies [45,46].

Traditional methodologies had to be transformed into student-centered ones to build learning through dialogue; without adequate teacher and student training, traditional or evidence-free methods prevail [42]. Participants in modern self-learning methods and PBL achieve significantly better results [18,19]. The student must be interested in managing the basic skills of a general practitioner, even if they are not in their favorite domain [45]. Reform is a necessity, but who will train the staff? Who ensures program quality with an objective perspective? [47] Channels for international communication such as seminars or training can be key points to changing the perspective of administrators, teachers, and students [41]. The evaluation is not only necessary but a responsibility insured by the faculties [21].

Results

Background: Honduras

Honduras is a country with 10.4 million inhabitants with a very low standard of living in position 135 of 196 countries [48], and the Human Development Index indicates a poor quality of life [49] and a hopeful life span of 62.8 years [50].

The current eight-year curriculum of the UNAH culminates in the Doctor of Medicine and Surgery (MD) degree [51]. In the first year, they reinforce general subjects [51]. Subsequently, in the second and third years, they begin with morphological sciences, physiological sciences, genetics, microbiology, and public health [51]. The fourth year provides lectures in the preclinical area [51]. These classes are mostly taught through master classes by students or teachers accompanied by practical laboratories [51]. During the fifth and sixth years, they join the wards of the local public hospitals [51]. The seventh year, a rotating internship, consists of classroom assignments, theoretical classes, and shift schedules, sometimes with immediate rest afterwards [51]. At the end of the clinical rotation, they must take a theoretical exam and an oral exam [51]. During the eighth year, called social service in primary healthcare or hospitals, this practice began in 1955 by decree law number 83, published in “La Gaceta”, since the country did not have enough graduated doctors at the time [51].

In 1995, Dr. Jorge Fernández described deficiencies in medical education in Honduras during a workshop in 1993 between semiology teachers and members of the Health Educational Technology Unit (UTES), in which he discussed the lack of integration of knowledge [52]. Thirty-four years later, there is no evidence of strategies to reduce these deficiencies. The literature demands substantial changes in health systems, and the only way to achieve them is through drastic changes in medical education [5].

In 1995, a reform of the medical studies curriculum was done at the UNAH [51]. ​​Said changes were made through general guidelines with the freedom to search and send comments to a commission [51]. Participatory curriculum reform in Iran was similar, though it had medical education expert supervision [53]. Similarly, the current plan had a process of reorganization and systematization of execution approved in 2000 [54]. In 2023, the UNAH was accredited by the Mexican Council for the Accreditation of Medical Education (COMAEM) [55]. To date, the results of the self-assessment or the action plan have not been published, which is why, despite being an achievement, it is not possible for the authors of this review to offer an analysis of them.

Experiences in Other Countries

In the context of the broad need for curricular reform, some countries undertook various reform processes (see Table 2) [5,10,11,16,19,20,30,32,35,36,43,47,53,56-65].

**Table 2:** Experiences in curricular reform in medical education from other countries: results and conclusions. Refs. [5,10,11,16,19,20,30,32,35,36,43,47,53,56-65]

Country	Article title or description	Results and/or Conclusions	Participants	Year of study	Type of study	Authors and reference
Iran	Defining a competency framework: the first step towards competency-based medical education	In retrospect, some of the errors found by some reforms in Iran include: not including the perspective of general practitioners or the perspective of the country's patients in the changes implemented.	130 Faculty members	2014	Participatory approach	Mirzazadeh et al., 2014 [53]
	A Decade of Reform in Medical Education: Experiences and Challenges at Tehran University of Medical Sciences	Once the reform was implemented, there was too much complexity in the initiative due to the inexperience and instability of the leadership with the overreach of resources, which caused demotivation.	Faculty members and medical education experts	2018	Comprehensive evaluation of the program	Mortaz et al., 2018 [30]
Iraq	Medical curriculum reform in Iraq	The evaluation of the reform is based on standards from accredited medical schools in developed countries requesting continuous feedback from students and graduates.	The National Council for Accreditation of Medical Schools	2018.	Perception	Al-Mendalawi, 2018 [47]
Arab Emirates	A Five-Year Longitudinal Study of the Educational Environment in a Newly Integrated Medical Curriculum	After five years of reform, learning was perceived as more positive, teachers were moving in the right direction, academic self-perception was more positive, the environment was more positive, and social self-perceptions were not so bad.	178	2019	Prospective study	Shehnaz, 2019 [11]
Kuwait	Comparison of Traditional Lecture-Based and Problem-Based Curriculum	The reform began in 2005. The first step was the transition from traditional teacher-centered and lecture-based methods to student-centered, small-group problem-based learning methodologies.	160	2016	Cross-sectional descriptive study.	Zahid et al., 2016 [19]
China	Analysis of curricular reform practices in Chinese medical schools	Most medical schools in China separated theory, internships and practices, neglecting the “Global Minimum Essential Requirements.” Furthermore, many subjects, by overlapping content between subjects, became redundant. Which unnecessarily increased teaching hours, turning learning into a burden without sufficient clinical, research and professional training. No less important, the evaluation was limited to written exams with no training component.	76 publications from 49 different medical schools.	2014	Systematic review of the literature published between 2001 and 2010.	Huang et al., 2014 [13]
	Curriculum reform in Chinese medical schools: What have we learned?	Not only did student perception improve, as the majority of medical schools reported an improvement in their students' clinical skills (76%) and research skills (60%).	38 medical schools	2014	Qualitative approach questionnaire for authorities	Huang et al., 2014 [36]
	Medical education reform at Wuhan University, China: preliminary report of an international collaboration	After an exhaustive investigation into the perception of the curriculum of one of the medical schools at the time, both professors and students (80%) believed earlier contact with the patient was imperative, almost 50% requested a reduction in class time , 70-90% suggested small group activities, clinical case discussions, and the opportunity for independent learning	296	2013	Student survey	Sherer et al., 2013 [56]
	Positive impact of integrating histology and physiology teaching in a medical school in China	After the implementation of the reformed curriculum in a hybrid model for international students, research was conducted that revealed that compared to their peers in the traditional curriculum, students in the reformed plan had a learning experience considered more favorable with the ability to see interdisciplinary links more clearly since integration reduced the redundancies that exist. Innovations such as integrating morphological sciences with physiological sciences and a greater emphasis on clinical application improve student participation with an active role in their learning, learning satisfaction, greater interdisciplinary relationship, greater interest, greater retention of learning.	243	2014	Student survey	Sherer et al., 2014 [57]
	Learning Effectiveness and Satisfaction of International Medical Students: Introducing a Hybrid-PBL Curriculum in Biochemistry	It should also be considered that the improvement was not only subjective, since with hybrid Problem-Based Learning models in biochemistry, students obtained higher scores than students with conventional teaching methods.	189	2017	Cross-sectional survey of students	Yan et al., 2017 [58]
Laos	Medical education in Laos	A key step implemented by medical schools was to change the evaluation system after realizing that exams with multiple choice questions were not enough, they required Objective Structured Clinical Examinations to evaluate competencies. To receive their degree, students must pass two final exams, their individual subjects, their research thesis, and have demonstrated professionalism during the period of their studies. Laos attributes much of the success of the reform to relationships with international experts as technical support for the educational reform that allowed the capacity of local personnel to be developed, the licensing exam to be developed meeting quality standards, and the evaluation process to be standardized. curriculum reform according to the World Federation for Medical Education.		2019	Description of important historical events	Wittick et al., 2019 [59]
Pakistan	Medical teachers’ perceptions of the integrated curriculum: a qualitative study	Despite not yet having quantifiable results of implementation, Pakistan conducted research on the current curriculum and how it can be improved, finding that all teachers believe that teacher training is essential before starting an integrated curriculum.	12	2018	Qualitative study from a teaching perspective	Kayani et al., 2018 [43]
Austria	A scientific approach to medical curriculum reform.	Resistance to curriculum reform in Vienna was inevitable, but not intransigent. It went from requiring small changes, to being impossible to achieve, to being necessary, but with greater preparation and planning, to becoming something inevitable in which even the toughest gave up resistance. A key piece, involving proactive adversaries in decision-making		2018	Description of important historical events	Marz, 2018 [35]
		Each existing department would maintain its working hours and salary, but the content, formats and curriculum were to differ. Although it seems trivial, this reduced uncertainty, and allowed openness to change. An interdisciplinary project approach was promoted in basic sciences, clinical teaching, curriculum development and even designated students. The team met in weekly sessions to define learning objectives, teaching formats, evaluation and interpretation of favorable results. Then, through sessions, seminars and master classes, the objectives and contents of the teaching were taught. Once implemented, feedback was systematically collected with proposals for improvement for the revision of the module.				
		Two programs were executed in parallel, the traditional one and the reformed one. This allowed us to compare and contrast results from both. For example, both groups had similar expectations of the program, but only the reformed pilot program reported feeling more prepared to practice. Furthermore, for objective results, students with the new curriculum obtained better results on medical progress tests compared to students with the traditional curriculum.				
Poland	Medical education in Poland	Part of the reform was the implementation of Structured Objective Clinical Examinations, clinical observations for the evaluation of professional behaviors		2019	Description of important historical events	Janczukowicz, 2013 [60]
	How does preclinical laboratory training influence physical examination skills during the first clinical year? A Retrospective Analysis of Objective Structured Clinical Examination Scores Routinely Collected Between the First Two Matriculation Classes of a Reformed Curriculum at a Polish Medical School	One of the most notable findings from students in the 2013 reformed curriculum was that OSCE Physical Examination scores were higher than those of students who participated in the previous 2012 curriculum.	807	2017		Świerszcz et al., 2017 [61]
Germany	The reformed Brandenburg medical curriculum: study local, work local	At a faculty in Brandenburg they took the initiative to transform the curriculum to one focused on practice, on the student, on science and on competencies from an interdisciplinary approach. The reform goes beyond clinical knowledge with a focus on personalized, patient-centered care, communication skills and personal development with a holistic perspective on social, economic, psychosocial and ethical issues.		2019		Winkelmann, 2019 [32]
		Although the focus is directly biomedical, natural science tutoring is offered to students with deficiencies in their previous secondary education. On the other hand, the rest of the content is covered in clinically defined modules with Problem-Based Learning sessions, master classes, practical approaches, diagnostic and therapy exercises, reflective practice space, interprofessional communication workshop, extracurricular courses, community services, elaboration of a research and scientific poster				
		The weekly structure of the theoretical program is somewhat complex, the authors describe it as follows. The Problem Based Learning case is related to the module of the week. During the session, learning objectives are worked on based on the student's self-directed learning with space to reflect in a second session at the end of the week. Pathologies are usually addressed by patients in settings (outpatient/hospital) or different specialties (surgery/internal medicine). The master classes are taught by teachers of basic sciences and clinical subjects, they are not mandatory, but attendance is almost perfect				
		They make use of practical single-subject courses ranging from anatomy to pathology and microbiology from a clinically relevant point of view. Another day of the week they carry out skills workshops such as anamnesis, physical examination, ultrasound or laboratory or imaging tests. Competencies on teamwork, humanity, reflection, interaction and communication are discussed in small groups throughout the semester. Interprofessional communication is carried out with the simulation of medical records, addressing everything from prevention and health promotion to shared decision-making. Likewise, every two weeks starting in the second year, students observe clinical practices and integrate into the hospital or community routine. At the end of the semester, the student is responsible for submitting a scientific article (on humanities in medicine or non-medical topics) with a poster that is presented at a conference and evaluated by experts. Students are also encouraged to participate in community work according to defined criteria and their own interests.				
		Student evaluation is carried out through written exams with questions that have been written in an exhaustive review process and through Objective Structured Clinical Examinations that evaluate practical skills and communication.				
Netherlands	Training medical students for the 21st century: Rationale and development of the Utrecht “CRU+” curriculum	The qualities to highlight were independent work, problem solving, teamwork and the ability to reflect on their actions before themselves and superiors.		2018	Description of important historical events	Ten Cate et al., 2018 [62]
Australia	Updating medical school psychiatry curricula to meet projected mental health needs	In Australia, multiple curriculum reforms were carried out. Among them, one carried out specifically for the teaching of psychiatry and related fields stands out since it was found that the quality and quantity of learning in these areas were insufficient to satisfy the anticipated mental health needs. The reform was based on basic competencies in the area, prevention, early intervention and mental health in health personnel. The study concludes with the express satisfaction that with the changes made, the field worked on will be adequately represented in the study plan.		2015	Bibliographic review	Thomas, 2015 [16]
Colombia	Medical education and the health system: why is it necessary to reform the curriculum?	By recognizing the need to integrate knowledge, Colombia managed with the new curriculum to train agents of change in the health system that positively affect communities. Thus achieving a transformation based on learning in a country with limited resources		2014	Description of important historical events	Quintero, 2014 [5]
USA	Fourth-year curriculum reform as a springboard for undergraduate medical training: experiences and lessons learned from one school	The first step was to find the deficiencies and from them develop a plan to reform the curriculum	152	2016	Qualitative summary of thematic analysis	Wackett et al., 2016 [63]
	Integration of clinical anatomical sciences in medical education: design, development and implementation of strategies.	Strategies for preparing material in a reformed curriculum include preparation (reading), linking (facilitating learning), engagement (providing relevance, guidance, discussion, and reflection), and transfer (applying knowledge obtained in future situations).		2021	Bibliographic Review	Khalil et al., 2021 [10]
		During the preparation phase, learning milestones were developed for integrated competency-based courses. Since early clinical exposure is the key to increasing confidence and preparation, courses such as biochemistry and anatomy were disbanded as the content was dispersed over five semesters in the form of early clinical experiences.				
	Learning by doing and shared discovery curriculum creation	Faculty involved in the classes that were modified were requested to generate content, grade assignments, or were trained to provide student-centered learning services.		2023	Description of important historical events	Sousa et al., 2023 [64]
		In conclusion, reform is considered a complicated process that takes time but is undoubtedly satisfactory.				
	Relationships Matter: Improving Trainee Development with a (Simple) Internship Curriculum Reform	Quantitatively, medical students in the new curriculum had more time with the same supervisors cultivating a degree of trust, so they performed more procedures than in traditional classes. Both students and teachers recognized the importance of having expected feedback competencies. The results of the surveys reported that the members of the traditional curriculum found it disorganized and confusing, while the participants of the reformed curriculum perceived it to be organized.	131	2019	Qualitative analysis	Dorsey et al., 2019 [45]
Canada	Reform of a radiology elective for final-year medical students through a needs assessment	In some cases, the reform begins or focuses on isolated subjects, in this case, radiology. It began with an assessment of learning needs to have a clear idea of ​​where the reform should be directed. Space for self-directed learning, discussions with residents, and space during the medical visit about radiological findings were described as activities that enhance the educational experience.	35	2018	Retrospective interview	Larocque et al., 2018 [65]
Haiti	The Haiti Medical Education Project: development and analysis of a competency-based continuing medical education course in Haiti through distance learning	Having one of the highest poverty rates on the continent has not been an excuse to stop the reform. They have managed to identify specific areas of competencies recommended for general practitioners that were not necessarily covered with traditional methodologies.		2016	Description of important historical events	Battat et al., 2016 [20]

Discussion

Deficiencies of the Current Medical Curriculum: Honduras

There is no evidence of a systematic evaluation of the curriculum. We must avoid making the mistake of identifying the inadequacy of the model and doing nothing to change it [66]. A culture that looks for deficiencies created by prolonged inattention must be promoted [47,67].

The unweighted use of traditional curriculum prioritizes the recall of information instead of integrating the knowledge necessary for your clinical practice [12,22,36,68,69]. In the fourth year, some teachers promote the “Diagnostic March” as a tool to integrate knowledge based on the manual “Surgical Diagnostic Teachings” published in 1986 by Dr. Silvio Zúniga, who warns at the preamble that it does not include bibliographic references since “there are none” about the topic [70]. Additionally, the opinion article “The Difficult Art of Teaching Surgery” published by Alejandro Membreño in 2010 is used [71].

There is no evidence of updating teaching methodologies. The benefits of PBL have been widely documented as a tool to integrate knowledge [2,5,9,10,13,15,18,19,24,26,30,32,36,38,41,44,58,60,64,72-82]. To date, there is no study, bibliographic review article, systematic review, letter to the editor, or comment that speaks of the benefits offered by the “Diagnostic March” over PBL methodology to justify its application.

There is no evidence of the use of effective feedback or standardized assessment methodologies. Marz pointed out that the use of inappropriate methodologies, little supervision, and feedback lead to suboptimal clinical skills [35]. There is no evidence of objective periodic assessment for minimum practical skills. Karami pointed out that the lack of this type of exam worsens the situation [23], abandoning the possibility of improving the performance of those involved [68].

The overload of patients in relation to medical personnel is irrefutable: seven doctors/100,000 inhabitants (when the WHO recommends at least 15) and 0.4 beds/100,000 inhabitants (when the WHO recommends at least 2.5) [83]. Due to the overload of patients, and the lack of qualified teaching and healthcare personnel, the student sometimes finds himself at the impasse of carrying out more bureaucratic tasks than academic or clinical tasks [61]. Studies in other countries with mandatory service in rural areas have shown that the majority of those who offer medical services do not perceive self-confidence; they also report uncertainty about when and who to ask for help due to little supervised clinical experience or the lack of feedback [68].

There is no evidence of activities that encourage reflective practice or self-directed learning by the student body. Promoting learning and reflection on experiences and actions as a future doctor is essential [29]. It is necessary to expand skills and include experience and training in communication and teamwork [4].

Strategies to Overcome Barriers and Challenges

Once it is accepted that a curriculum can be improved, reform is not only necessary but must emerge in a revolutionary way [35]. Curriculum reforms usually follow six steps: problem identification, needs assessment, expected results, training strategies, reform development and implementation, and evaluation and feedback [23,25,84]. It is recommended to implement the reform in two phases: first with a limited number of students and then with the support of experts [79].

(1) Identification

Basic required improvements: Faculties and authorities must commit to honest scrutiny of their current program to develop adapted strategies and be successful [37]. They can create a questionnaire like some medical schools in China did [36] or use the “Dundee Educational Environment Readiness Measure (DREEM) Questionnaire” to determine the nature of the problem through an assessment of the needs of all entities before developing curricular changes like the Arab Emirates and other China schools did [11,53]. In China, various faculties have discovered that only half of the staff consider the curriculum satisfactory [53].

Effective curricular reform cannot be promoted without a profile of clear student competencies aligned with the needs of the country [22]. Competencies should be broken down into standards to facilitate feedback [21]. The "Swiss Medical Interfaculty Commission'' (SMIFK) includes the “Main Relevant Objectives for Learning and Integrated Education in Switzerland” (PROFILES) and the “Swiss Catalog of Learning Objectives for Undergraduate Medical Education” [75,85]. Some reforms began with national laws that demanded curricular changes based on these documents, adapted to national conditions [73].

(2) Training

It has been studied in China that students in student-centered curricular models perceive more abilities, but rate themselves as having less medical knowledge than their peers in conventional models [58]. To optimize performance, it is essential to ensure timely, continuous, and effective training of all personnel involved [41]. Understanding that academic freedom entails responsibility, it is necessary to strengthen teachers towards modern teaching and evaluation methods [79]. The role of the teacher is fundamental, acting as a role model in the clinical and non-clinical environment, and serving as a source of reflection and feedback [29,31].

Curriculum reform implies a change in instruction methods for which teachers require training according to China's experience [36]. There is greater resistance in teachers than in students to new methodologies [79]. PBL helps with clinical competencies but is not recommended for theoretical knowledge, as Kuwait suggests [19]. Master classes taught by trained teachers are essential and must be complemented by PBL [82]. In the United States of America (USA), for subjects such as anatomy, hybrid models with self-directed learning and dissection workshops are recommended [10]. The evidence that students in models integrated with PBL obtain better scores and develop diverse skills should be highlighted as shown by Kuwait and China [19,58,66].

Multiple choice questions objectively, reliably, and validly assess basic cognitive skills in the USA [19]. However, they can be superficial and trivial, ignoring deeper competencies, and highlighting the need to implement other evaluation methods [81]. Formative assessment provides continuous feedback on student progress, correcting errors in patient care, behavior, professionalism, and communication in China [36]. The best way to document this progress is through the use of the portfolio [27]. Timely and exceptional teacher feedback is vital to transforming a passive and stressed student into a self-directed and outstanding one. It is the cornerstone of clinical teaching and must be based on a standardized, not subjective, objective map of competencies [22]. In Poland, among the benefits of using Observed Structured Clinical Examinations (OSCE) is the ability to predict future performance in the clinical setting [61]. Comprehensive OSCEs are essential to assess practical skills, where multiple-choice questions are not sufficient [30]. To corroborate the improvements of the curricular reform, OSCE results can be compared between both curricula [86].

Kuwait showed that self-directed learning combines prior knowledge that is increasingly refined and perfected [19]. PBL sessions and lecture-based learning, as Iran implemented, should encourage the student's reflective practice, questioning the process until they understand it through teamwork, leadership, and communication [30]. Effective feedback depends on the student's reflection and action [22]. Recording in portfolios is introduced in the curricular reform to actively engage in humanistic and moral training with exemplary teaching models [27].

(3) Involve strategic participants

If resistance, power, and ego struggles are inevitable, as the Arab Emirates and Iran experienced; the only way to carry out successful reform is a strategic participatory approach [11,25,53]. The logical order indicates that change agents must first be chosen from those who understand the purpose of the reform like China did [13,72,78,79]. It is noted that one of the challenges is the organizational structure of traditional departments as mentioned by USA [10]. Nor should we make the mistake of involving only experts without the interested parties understanding the long-term vision [28,87].

Curricular reform with an integrated competency-based approach irrefutably depends on discernment, expertise, and synthesis capacity provided only by experts as China adverts [13,21,41,56,76]. Iran says is advisable to realize teaching collaboration from all departments [30]. To ensure the quality with which students, and future doctors, acquire their learning and care for patients, it must be ensured that their perspective is in line with the workflow since they are the users and even collaborators of the methodologies that will be implemented like Iran warned [30,88]. Laos said that applying OSCE systematically, introducing stress management techniques, and adding electronic tools for recording feedback and progress are bureaucratic aspects that must be managed by the corresponding administrators [59].

(4) Development and implementation of the new study plan

Suggested plan: A suggestion of the curriculum adapted to the current health needs of the country can be drawn from integrated models and experiences of curricular reform in other countries such as subjects and rotations (see Table 3), methodologies and tools (see Table 4), and mastery level and evaluation methodologies adapted by those implemented by Arab Emirates, Iran, and Laos (see Table 5) [11,22,30,51,59,76,89]. The cornerstone of a competency-based curricular reform is to align the student's knowledge and skills to their level of responsibility [22]. The scale, structure, and design of medical training must be constantly improved according to the needs of the health system to deliver doctors whose level of mastery of skills have been verified to practice as a general practitioner in their country [69]. Identifying your own competency framework reinforces the sense of ownership [53]. This also makes it easier for every activity, every learning, every evaluation, and every feedback to be located within said organizing framework to refer to it [22]. To facilitate learning, it is useful, according to Iran, that the final model with the expected competencies should be transmitted to teachers and students to ensure that they know what results are expected of them when teaching and learning respectively [53].

**Table 3:** Subjects and rotations suggested for curricular reform. The duration of the rotations with the systems approach will be adjusted to their content. Adapted from the current health needs of the country, integrated models and curricular reform experiences in other countries [11,22,30,51,59,76,89].

Year of the training	1	2	3	4	5	6
	Basic sciences	System-based learning	Clinical medicine	Clinical placement	Clinical placement	Internship
Subjects	IDEM Current Study Plan	Anatomy Histology Embryology Genetics Physiology	Pathology Pathophysiology Pharmacology Semiology	Family Medicine Internal Medicine Surgery Pediatrics obstetrics gynecology Trauma Radiology Epidemiology	Family Medicine Internal Medicine Surgery Pediatrics obstetrics gynecology Trauma Radiology Epidemiology	Family Medicine Internal Medicine Surgery Pediatrics Gynecology and Obstetrics
Main focus		Clinical relevance	Knowledge integration	Emergency Medicine	Primary Health Care Legal and forensic aspects Administration and management	Specialty specific complications
Placements						
Cardiovascular system				Cardiology Vascular surgery	Cardiology Vascular surgery	Cardiology Vascular surgery
Hematopoietic system				Hematology	Hematology	Hematology
Respiratory system				Pneumology ENT	Pneumology ENT	Pneumology ENT
Digestive system				Gastroenterology	Gastroenterology	Gastroenterology
Endocrine system				Endocrinology	Endocrinology	Endocrinology
Urinary system				Urology	Urology	Urology
Urinary system				Nephrology Urology	Nephrology Urology	Nephrology Urology
Nervous system				Neurology Psychiatry	Neurology Psychiatry	Neurology Psychiatry
Special senses				Neurology Ophthalmology ENT	Neurology Ophthalmology ENT	Neurology Ophthalmology ENT
Immune system		Microbiology		Immunology Infectology tropical diseases	Immunology Infectology tropical diseases	Immunology Infectology tropical diseases
Musculoskeletal system				Rheumatology Orthopedics	Rheumatology Orthopedics	Rheumatology Orthopedics
Integumentary system				Dermatology	Dermatology	Dermatology
Basic courses			First aid Invasive and non-invasive procedures		Intermediate Life Support (ILS)	

**Table 4:** Suggested methodologies and tools. SDL: Self-Directed Learning; PBL: Problem-Based Learning; IDM: Integrated Didactic Masterclasses; S: Simulation; SCP: Supervised Clinical Practice Adapted from the current health needs of the country, integrated models and curricular reform experiences in other countries [11,22,30,51,59,76,89].

Year of training	1	2	3	4		5		6	
	Basic sciences	System-based structure	Clinical medicine	Clinical placements		Clinical placements		Internship	
Methodologies									
Portfolio – Formative evaluation (Training workshop, simulation, reflection and feedback)		Bioethics Interpersonal communication	Bioethics Interpersonal communication		Bioethics Interpersonal communication Give bad news		Bioethics Interpersonal communication Give bad news Patient treatment		Bioethics Interpersonal communication Give bad news Patient treatment
Investigation		Thematic research	Project-based research				Thematic research		Thesis
Teaching methodologies		SDL IDM Dissection Laboratories	SDL PBL IDM S		SDL PBL IDM S SCP		SDL PBL IDM S SCP		SDL PBL IDM S SCP

**Table 5:** Suggested mastery level and evaluation methodologies. Adapted from the current health needs of the country, integrated models and curricular reform experiences in other countries [11,22,30,51,59,76,89].

Year of training	1	2	3	4	5	6
	Basic sciences	System based structure	Clinical medicine	Clinical placements	Clinical placements	Internship
Mastery level						
Clinic history		Distinguish physiological from pathological	Knows the content and order to follow during anamnesis and Physical Examination Recognize the typical presentation of the disease Describe the pathophysiology of the disease	Specifies important details of symptoms and signs Individualizes probable differential diagnoses	Conducts a directed interrogation and examination with positive, negative, and relevant findings	Identifies symptoms associated with complications at the level of your suspected diagnosis. List complications at your level
Interpretation			Know the common causes of the reason for consultation	Argue and support the most probable cause	Redirects its order and emphasis according to findings found Is able to summarize relevant clinical, laboratory and imaging findings	Knows when to refer or when to request support or consultations
Treatment			Knows the main exams and treatments for the most frequent causes of the reason for consultation	Argues and supports the need for an examination or therapeutic plan	You can detail a therapeutic plan at your level	Provides initial management at your level
Patient care			Friendly and professional	Allows the patient to ask questions and provides explanations	The care provided is individualized and empathetic	The care provided is individualized and empathetic
Professionalism		Recognize the roles and responsibilities of the doctor before society Demonstrate motivation through appropriate self-directed learning Develop reflective and critical thinking	Interpret the role of empathy with patients and respect for their preferences, beliefs, culture and state legal and ethical codes.	Demonstrate effective time and stress management skills Recognize ethics and professionalism as an integral part of the doctor-patient relationship.	Respond appropriately in the event of medical errors and understand their implications in the event of non-disclosure.	Communicate effectively. Demonstrate respect for the knowledge, skills and experience of other team members Know how to organize and lead a team
Ethics		Understand the principles of ethical practices applied to patient care, research, and medical education	Demonstrate professional behavioral attributes of patient respect, autonomy, consent, non-maleficence, and confidentiality when taking medical history and performing physical examination of patients.	Demonstrate a solid understanding of the principles of ethical decision making, Obtain informed consent and address patient/attending queries effectively	Maintain confidentiality and privacy in patient care	Identify and discuss issues related to abortion, medical termination of pregnancy and reproductive rights, organ transplantation and body donation, LGBTQ rights
Methodologies						
Suggested evaluation	Portfolio	Portfolio OSCE Knowledge Comprehension Written	Portfolio OSCE Knowledge Comprehension Practical and written	Portfolio	Portfolio OSCE Knowledge Comprehension Practical and written	Portfolio

(5) Comprehensive, periodic, and systematic evaluation

The evaluation process must have adequate and permanent committees, units, structures, and resources from the beginning of the reform to maintain and refine the development of the curriculum as Iran suggested [4,25,28,30,76]. On the other hand, the absence of comprehensive, periodic, and systematic evaluation compromises the vulnerability of the reform to being supported by anecdotal evidence and rumors, as the USA warned [10,41]. The evaluation committee within the faculty must be recognized by the accreditation body, the World Federation for Medical Education (WFME) [69].

The following steps of Kirkpatrick's evaluation model, recommended by reforms implemented in the USA, are student satisfaction; evaluation and interpretation of knowledge; perceived changes in ability; and measurable objectives [10]. The data revealed through continuous evaluation provide a clear image of the curricular reform and whether it is on the right path, or if it should be redirected [87].

Canada affirms that the teacher is the main issuer of the reform to recognize if it has achieved the expected vision, student feedback is paramount as it provides insight into the effectiveness of teaching strategies [65]. Visits from a non-program perspective by national and international consultants, especially delegates from the Ministries of Health and WFME, are encouraged to be scheduled [30]. If possible, feedback should be sought from professionals who interact with the student and medical graduate, as well as policymakers within the government [2,68,69,90]. Given that the government is primarily responsible for financing the education and health of its population, it is expected that it will be interested in the findings of the evaluation process and, even through this, encourage long-term financing of the reform [41,87].

## Conclusions

The need for a comprehensive curricular reform in the UNAH medical career is theoretically and empirically enormous. The experience of other countries in similar conditions reinforces the need to carry out reform, regardless of the country's state of development. There are important challenges that can be overcome through viable strategies and recommendations that help adopt a change in the curriculum that involves knowledge integration and student-centered and competency-based active learning, without forgetting the essentials of patient-centered care. To achieve the changes that are expected to be seen in the health system, one of the first steps to consider should be to deliver professionals trained with methodologies that have collected evidence of improvements in academic and work performance with a high impact on humanly favorable evolution from the patients. The key to success will be based on the identification of change agents with a clear vision of the minimum competencies expected of a general practitioner, updated and timely training, and the linking of all the necessary parties from the beginning to the implementation, without forgetting the key step of continuous evaluation.
